# The role of generative AI in facilitating children's creative thinking and cognitive development: a systematic scoping review

**DOI:** 10.3389/fpsyg.2026.1880052

**Published:** 2026-09-14

**Authors:** Tianyue Niu, Huiying Liu, Patrick Pang, Yiming Taclis Luo, Ting Liu

**Affiliations:** 1 School of Design, Jiangnan University, Wuxi, China; 2 Xiamen Academy of Arts and Design, Fuzhou University, Xiamen, China; 3 Jimei University, Xiamen, China; 4 Faculty of Applied Sciences, Macao Polytechnic University, Macao, China

**Keywords:** children, cognitive development, creative thinking, GenAI, generative AI, systematic scoping review

## Abstract

**Background:**

Generative Artificial Intelligence (GenAI) has reshaped STEAM education, with growing interest in its use to enhance children's creative thinking a key cognitive development component. However, systematic synthesis of GenAI's application in child-centered creative learning remains limited. This scoping review explores GenAI's roles, effects, challenges, and future directions.

**Methods:**

This study follows JBI guidance and is reported in accordance with PRISMA-ScR. Searches were conducted in July 2025 and updated in May 2026 across Web of Science, Scopus, ERIC, APA PsycInfo, ACM Digital Library, ProQuest, and Google Scholar. Publication status and evidence-source type were charted explicitly.

**Results:**

Twenty-four evidence sources were included: 22 primary empirical studies and two secondary syntheses used only for contextual interpretation. Text-based LLMs and multimodal text/image tools predominated. The primary studies reported possible benefits for divergent thinking, narrative creativity, and creative self-efficacy, but the heterogeneous designs, absence of formal critical appraisal, and inclusion of preprints preclude conclusions about confirmed efficacy.

**Conclusion:**

The emerging evidence suggests that carefully facilitated GenAI may support some forms of children's creative activity, but the evidence base is small, methodologically heterogeneous, geographically concentrated, and partly non-peer-reviewed. Claims about effectiveness, scalability, and equity therefore remain provisional. Future research should use stronger comparative and longitudinal designs and directly include rural, underserved, and low-resource communities before GenAI is presented as a democratizing intervention.

## 1 Introduction

Children represent the primary foundation of future intellectual capital and social innovation, and their developmental trajectory holds profound significance for both individual wellbeing and long-term societal progress (Roy and Sahoo, 2025). In the 21st-century global education landscape, STEAM (Science, Technology, Engineering, Arts, and Mathematics) education has emerged as a pivotal framework for cultivating innovative, creative, and interdisciplinary thinkers (Papadopoulou, 2024). Unlike traditional STEM (Science, Technology, Engineering, and Mathematics) education, STEAM integrates the Arts as a core component, emphasizing creative expression, cultural relevance, and aesthetic reasoning alongside technical and scientific rigor (Belbase et al., 2022). Within this trajectory, creative thinking, defined as the cognitive ability to produce ideas that are both novel and task-appropriate, serves not only as an elective artistic skill but as a core prerequisite for ensuring a child's cognitive function, academic problem-solving, and socio-emotional stability (Barr, 2014). In the early educational setting, robust creative thinking allows children to effectively navigate complex learning environments, enhances cognitive flexibility, and promotes a sense of agency (Valquaresma, 2024). From a neuropsychological perspective, creativity is intrinsically linked to the maturation of executive functions, including working memory, inhibitory control, and mental shifting (Benedek et al., 2014). These functions provide the necessary mental architecture for divergent thinking, the ability to explore multiple solutions to an open-ended problem (Zhu et al., 2024). However, the development of these skills is not automatic; it is a sensitive process influenced by the tools and environments children interact with (Deák, 2014). As the global educational landscape shifts toward “21st-century skills,” the early identification of creative potential and the implementation of sustainable support for cognitive growth have become crucial for mitigating the risk of developmental stagnation and ensuring that children can thrive in an increasingly automated and complex world (Ayber, 2024).

To address the creative and cognitive needs of children, traditional pedagogical models have long been dominant. These models primarily include classroom-based arts and crafts, play-based learning in physical environments, teacher-led brainstorming sessions, and the use of physical manipulatives like building blocks or drawing tools. While these traditional models are grounded in established developmental theories, such as Piaget's constructivism (O'loughlin, 1992), and have played a vital role in fostering early motor skills and imagination, they face multiple limitations when coping with the large-scale, diverse needs of modern global education (Duffy, 2006).

There is the issue of resource accessibility and geographical equity. Access to high-quality creative mentorship, such as specialized art teachers, music tutors, or creative writing experts, is often a privilege of high-income urban areas, leaving children in remote or underserved communities with limited exposure to diverse creative stimuli (Peppler et al., 2022). Traditional models often lack real-time personalization and scalability (Mourtzis et al., 2023). In a standard classroom, a single teacher struggles to provide immediate, individualized scaffolding for thirty different creative projects simultaneously (Sawyer, 2019). This means that a child's “creative spark” may be lost due to a lack of immediate feedback or the inability to iterate on an idea in real-time (Moore, 2017). Traditional formats can sometimes create psychological barriers to entry. Children who lack fine motor coordination, e.g., difficulty drawing or writing, may feel excluded from creative expression, leading to “creative anxiety” and a withdrawal from participation (Goodwin, 2023). These limitations, particularly the lack of high-precision personalization and the structural inequality of resource distribution, make traditional models insufficient for maximizing the creative potential of all children, highlighting the urgent need for innovative, scalable solutions (Bulathwela et al., 2024).

Against this background, GenAI is emerging as a critical driver transforming the children's developmental landscape due to its unique advantages in speed, breadth, and interactivity (Neugnot-Cerioli and Laurenty, 2024). GenAI represents a subset of AI characterized by the ability to create novel content ranging from text and images to audio and synthetic data that mimics human-like creativity (Di Biagio, 2022; Rajasekar and Subramaniyaswamy, 2025). Unlike traditional “Discriminative AI,” which focuses on classifying or predicting based on existing data, GenAI leverages foundation models and deep learning architectures (Luo Y. et al., 2025; Rahman, 2025), such as Transformers and Diffusion Models, to learn the underlying patterns of vast datasets and generate entirely new outputs based on user-provided prompts (Luo et al., 2026). In the developmental domain, this technology primarily manifests through large language models (LLMs) like ChatGPT (Luo et al., 2025), image synthesis tools such as Midjourney or DALL-E, and multi-modal co-creation platforms that integrate text, sound, and visual elements (Ko et al., 2023).

The core advantages of GenAI lie in its high accessibility, real-time feedback loop, and adaptive personalization, allowing it to remedy the flaws of traditional creative models (Mohamed et al., 2025). For instance, text-to-image generators allow children who may lack advanced fine motor skills to visualize their complex inner worlds simply by articulating prompts, thereby decoupling creative intent from technical execution (Hu X. et al., 2025). This non-invasive support allows children to see their ideas manifested instantly, increasing their intrinsic motivation and willingness to engage in iterative thinking (Jaquith, 2011). By integrating these AI-driven mechanisms into learning, such as collaborative storytelling or AI-assisted design, GenAI platforms have significantly boosted children's engagement (Han, 2024). Furthermore, these platforms provide a cost-effective way to deliver “expert” creative feedback to any location with an internet connection, effectively overcoming geographical constraints. These multi-faceted applications indicate that GenAI is not merely a simple alternative to traditional tools but an essential instrument for achieving creative exploration and large-scale personalized cognitive support, holding potential to change the paradigm of children's creative education (Moura and Moruzzi, 2026).

Despite the rapid and widespread integration of GenAI in children's environments, existing research and reviews often suffer from fragmentation and a lack of systematic integration, which limits their utility for evidence-based practice in education and public health. Some existing reviews exhibit the following limitations: firstly, most reviews concentrate on a single technology or a single broad outcome, e.g., general literacy, and this point-wise approach fails to offer a comprehensive, cross-technology perspective to understand how different GenAI modalities impact various dimensions of creativity, like fluency, flexibility, and originality (Karani and Mwancha, 2025). Secondly, there is a lack of systematic exploration of practical application and implementation details. Existing literature primarily focuses on the quantifiable output, but pays insufficient attention to how the technology is integrated into the child's workflow, the implementation barriers faced by educators, and the qualitative user experience of the children themselves. There is inadequate depth in discussing ethical, safety, and developmental challenges. With the deeper integration of GenAI, risks such as algorithmic bias that might limit a child's cultural perspective are becoming increasingly prominent.

To fill these gaps in the systematic and comprehensive scope of existing research, this study aims to conduct a thorough, systematic scoping review to map how GenAI has been studied in relation to children's creative thinking ability. Following JBI guidance and reporting in accordance with PRISMA-ScR (McGowan et al., 2020), this study will systematically search, screen, and synthesize literature on the intervention and application of GenAI in the pediatric creative and cognitive domain globally. The core objectives are to comprehensively identify the GenAI types currently applied to children; systematically analyze the reported roles of these technologies in promoting specific cognitive dimensions; and clearly define the technical, ethical, and practical barriers present in this field. This study will provide evidence-based guidance for educators, pediatric health experts, and technology developers, helping them select and design the most effective, safe, and age-appropriate GenAI solutions. Based on the above background and objectives, this study proposes five core research questions:

(1) What are the geographic and temporal trends in the application of GenAI to children's creative thinking, and what research methodologies are predominantly employed in this field?

(2) What types of GenAI technologies are currently utilized to support children, and what are the primary creative or cognitive domains they target?

(3) What are the common intervention forms of these GenAI applications, and what measurement indices are used to assess the reported effects on children's creative thinking?

(4) What barriers and challenges exist in the practical application of GenAI for promoting children's creative thinking?

(5) What are the key future research directions for GenAI in promoting children's creative and cognitive development?

## 2 Methods

This scoping review was conducted following the Joanna Briggs Institute (JBI, Munn et al., 2019)methodology for scoping reviews and is reported in accordance with the PRISMA-ScR checklist (Tricco et al., 2018.

### 2.1 Eligibility criteria

The eligibility criteria were defined using the PCC framework to ensure structural clarity and academic focus (Santana et al., 2018).

Population: the primary population of interest was school-aged children and young adolescents within the 6 to 15-year-old age range, encompassing students in primary and lower secondary education. This represents a critical developmental window for the maturation of executive functions and the cultivation of divergent thinking skills. However, given the developmental continuum of creative cognition and the limited availability of studies strictly confined to this range, studies that included children slightly outside this range. e.g., early childhood aged 5–6, or early adolescents aged 16–18, were considered for inclusion if the majority of the participant cohort fell within the target range, or if the study's findings were directly applicable to the 6–15 age group. This approach is consistent with scoping review methodology, which permits flexible boundary conditions to ensure comprehensive coverage of the evidence base. Each borderline case was explicitly reviewed and justified by the research team.

Concept: the core concept was the application of GenAI to support, monitor, or enhance creative thinking abilities. For this review, GenAI was operationally defined as tools or platforms leveraging large-scale datasets and deep learning architectures to generate novel content. This includes text-to-text generators, text-to-image synthesis, and multi-modal co-creation tools. Creative thinking outcomes were defined as measurable cognitive processes related to problem-solving and ideation, focusing on dimensions such as originality, fluency, flexibility, and elaboration. Studies focusing on non-generative AI or those lacking a clear link to creative outcomes were excluded.

Context: the context refers to any setting where students engage with GenAI, encompassing both formal academic environments, e.g., primary/secondary school classrooms, ICT labs, and informal learning contexts, e.g., after-school workshops, home-based learning, and museum-led AI programs.

The core evidence set comprised English-language primary empirical studies (e.g., randomized or quasi-experimental studies, case studies, participatory/user studies, surveys, and qualitative research) with full text available. Two secondary syntheses (Ali et al., 2024; Pérez-Jorge et al., 2026) were retained as a prespecified contextual exception because they synthesized implementation and ethical issues across the target educational context. These secondary sources were labeled separately, were not counted as primary empirical evidence, and were not used as independent confirmation of study-level effects. Peer-review status was not an eligibility requirement; peer-reviewed publications, conference papers, institutional reports, and openly available preprints were eligible when all substantive PCC criteria were met. Publication status was charted and considered when interpreting evidentiary strength. Studies focused solely on technical model development, algorithm comparison without child application, or adult perceptions without a link to children's creative outcomes were excluded. The final inclusion and exclusion criteria are summarized in Table 1.

**Table 1:** Inclusion and exclusion criteria.

Inclusion criteria	Exclusion criteria
I1. Participants are school-aged children or young adolescents (core target: ages 6–15); borderline cases (ages 5–6 or 16–18) may be included if the majority of participants fall within the target range or findings are directly applicable.	E1. Participants are exclusively preschoolers (< 5 years) or adults (>18 years), with no overlap with the target 6–15 range.
I2. The study investigates GenAI (e.g., LLMs, image synthesis, multi-modal GenAI).	E2. The study focuses exclusively on non-GenAI or traditional digital tools.
I3. Primary evidence reports empirical data on children's creative-thinking outcomes; up to two clearly labeled secondary syntheses may be retained only for contextual interpretation.	E3. The study lacks a primary focus on creativity (e.g., focusing only on general academic grades).
I4. Primary empirical research (e.g., trials, quasi-experimental, survey, case, participatory, or qualitative studies); publication status is charted.	E4. Editorials, opinion pieces, or purely conceptual frameworks; secondary studies without unique contextual relevance.
I5. The study explores actual field applications or interventions with children.	E5. The study is limited to technical algorithm development or laboratory testing without child interaction.
I6. Full text available in English; peer-reviewed and eligible non-peer-reviewed sources are labeled separately.	E6. Full text not available or published in languages other than English.

### 2.2 Search strategy

A comprehensive search strategy was developed and executed in July 2025, with a supplementary update in May 2026. Seven multidisciplinary sources were searched: Web of Science, Scopus, ERIC, APA PsycInfo, ACM Digital Library, ProQuest, and Google Scholar. Three Boolean concept blocks covered (1) GenAI, (2) the child/school population, and (3) creativity. The same conceptual logic was retained across sources, while syntax was adapted to each interface. Searches were restricted to title, abstract, and keywords where the database supported consistent field searching; all searchable fields were used where equivalent field restrictions were unavailable. Google Scholar was used as a supplementary broad-discovery source. Its query was explicitly parenthesized, the search and update dates were recorded, and all records were deduplicated before screening. Because Google Scholar contributed 3,859 of the 6,401 initial records and its ranking/indexing are dynamic, this source is treated as a reproducibility limitation rather than equivalent to a controlled bibliographic database. Full database-specific strings are reported in Table 2.

**Table 2:** Selected databases and search queries.

Database	Search query
Web of Science	(“Generative AI” OR “GenAI” OR “Large Language Model” OR “LLM” OR “ChatGPT” OR “text-to-image” OR “DALL-E” OR “Midjourney” OR “stable diffusion” OR “co-creation AI”, All fields) AND (“children” OR “pediatric” OR “preschool” OR “student” OR “adolescent” OR “minor”, All fields) AND (“creativity” OR “creative thinking” OR “divergent thinking” OR “originality” OR “imagination” OR “problem solving”, All fields) AND (Document Types: Article or Proceeding Paper) AND (Languages: English)
Scopus	[TITLE-ABS-KEY (“Generative AI” OR “GenAI” OR “Large Language Model” OR “LLM” OR “ChatGPT” OR “text-to-image” OR “DALL-E” OR “Midjourney” OR “co-creation AI”)] AND [TITLE-ABS-KEY (“children” OR “pediatric” OR “student” OR “adolescent”)] AND [TITLE-ABS-KEY (“creativity” OR “creative thinking” OR “divergent thinking” OR “originality” OR “problem solving”)] AND [LIMIT-TO (DOCTYPE, “ar”) OR LIMIT-TO (DOCTYPE, “cp”)] AND [LIMIT-TO (LANGUAGE, “English”)]
ERIC	TI, AB(“Generative AI” OR “Generative Artificial Intelligence” OR “Large Language Models” OR “ChatGPT” OR “Text-to-Image”) AND TI, AB(“elementary education” OR “secondary education” OR “middle school” OR “primary school” OR “grade 1–9”) AND TI, AB(“creative thinking” OR “creativity” OR “divergent thinking” OR “imagination”)
ACM Digital Library	[(All: “generative ai”) OR (All: “generative artificial intelligence”) OR (All: “chatgpt”) OR (All: “llm”)] AND [(All: “children”) OR (All: “students”) OR (All: “middle school”)] AND [(All: “creativity”) OR (All: “creative thinking”) OR (All: “co-creation”)]
APA PsycInfo	(Anywhere: “Generative AI” OR “ChatGPT” OR “Large Language Model”) AND [Age Group: “School Age (6–12 yrs)” OR “Adolescence (13–17 yrs)”] AND (Anywhere: “creativity” OR “creative thinking” OR “divergent thinking” OR “problem solving”)
ProQuest	TI, AB(“Generative AI” OR “ChatGPT”) AND TI, AB(“children” OR “schooling”) AND TI, AB(“creative thinking” OR “creativity”)
Google Scholar	(“Generative AI” OR GenAI OR ChatGPT OR “large language model” OR “text-to-image”) AND (child OR children OR school OR student OR adolescent) AND (creativity OR “creative thinking” OR “divergent thinking” OR originality OR imagination OR “problem solving”) [all searchable fields; searches run July 2025 and updated May 2026]

### 2.3 Study selection and data charting process

All identified records were exported to EndNote, where 2,528 duplicate records were removed before screening. Two reviewers (H.L. and Y.T.L.) independently screened titles and abstracts against the eligibility criteria. Reports considered potentially relevant were sought in full text and assessed independently by the same reviewers. Reasons for exclusion were recorded at full-text assessment. Disagreements were resolved through discussion or consultation with a third reviewer (T.L.).

A standardized data charting form was developed and piloted on a sample of five included studies. The form captured: author(s) and year; country; study aims; methodology; participant characteristics (age, setting); type of GenAI used (text, image, etc.); intervention form; targeted creativity dimensions; measurement indices; key findings; reported barriers, e.g., ethical concerns, cognitive dependency; and conclusions. Data charting was performed independently by two reviewers, with interrater reliability calculated using Cohen's kappa (κ), achieving substantial agreement for study selection (κ = 0.812) and data charting (κ = 0.778).

### 2.4 Collating, summarizing, and reporting the results

The charted data were analyzed using descriptive mapping and narrative synthesis, as appropriate for a scoping review. Primary empirical studies formed the basis of the study-level findings; the two secondary syntheses were used only to contextualize implementation and ethical themes and were not double-counted as independent evidence of effects. Quantitative findings were summarized descriptively, without pooling or effect-size estimation, because of substantial heterogeneity. No formal risk-of-bias or methodological quality appraisal was undertaken; consequently, the synthesis describes reported associations and outcomes rather than establishing efficacy. Publication status was charted, and potential selective publication and positive-results bias were considered when interpreting the direction of findings.

## 3 Results

### 3.1 Search results

The searches identified 6,401 records. After 2,528 duplicates were removed, 3,873 unique records underwent title/abstract screening; 3,746 were excluded, and 127 reports were sought for retrieval. Fifty-eight reports could not be retrieved, leaving 69 reports for full-text assessment. Forty-five were excluded: no primary focus on creativity (*n* = 22), use of non-GenAI or traditional digital tools (*n* = 13), no direct child interaction/application data (*n* = 8), and ineligible publication or study type (*n* = 2). Twenty-four evidence sources werei ncluded: 22 primary empirical studies and two secondary syntheses retained for contextual interpretation (See Table 3). These counts close at every stage and are shown in Figure 1.

**Table 3:** Characteristics of the included studies.

Author/year/ country	Study aims	Methodology (design)	Target cohort (age/grade)	Setting	GenAI type/ modality	Intervention form & duration	Targeted creativity dimensions	Measurement indices/ instruments	Key findings & reported barriers
Newman et al., 2024 USA (Newman et al., 2024)	How GenAI impacts children's creative self-efficacy	Participatory Design (Cooperative Inquiry)	12 children	Non-formal (Design workshops)	Text (ChatGPT), Image (DALL-E 2), Music (Magenta.js)	6 workshops, 90 mins each over 4 months	Creative self-efficacy, Divergent thinking	Qualitative (artifacts, video, inquiry notes)	Findings: Iterative prompting builds mental models; GenAI aids divergent thinking Barriers: Formal system language, lack of transparency, ethical/authorship concerns
Ali et al., 2024 Bangladesh (Ali et al., 2024)	The impact of GenAI on learning and creativity among Gen Z students.	Secondary literature review [contextual evidence; not a primary empirical study]	Generation Z students (born late 1990s to early 2010s)	Formal education and creative industries	Text, image, music, and code (e.g., ChatGPT)	Theoretical analysis of AI integration in curricula and creative workflows	Creative thinking, artistic expression, and democratization of creativity	Qualitative synthesis of academic publications, industry reports, and case studies	Findings: GenAI personalizes learning, provides instant feedback, and enhances artistic inspiration Barriers: Risks of diminished critical thinking, algorithmic bias, data privacy, and copyright/authorship issues
Fitriyani et al., 2025 Indonesia (Fitriyani et al., 2025)	To develop and test GenAI-enhanced Game-Based Learning (GBL) to improve student creativity in Science.	Research and Development (RnD) using the ADDIE model	20 5th-grade elementary students (approx. age 10–11)	Formal education (Elementary school)	Text-based (ChatGPT-3.5 API) integrated into a game platform	Adaptive game scenarios and challenges generated by AI based on student responses	Originality, fluency, flexibility, and elaboration (via Pre-test/Post-test)	Quantitative (N-gain score) and observation of creative products	Findings: Significant improvement in creativity (N-gain score 0.76, “High” category); AI successfully personalized game challenges Barriers: Need for stable internet connectivity; requires teachers' technical readiness to facilitate AI-GBL
Pérez-Jorge et al., 2026 Spain (Pérez-Jorge et al., 2026)	To synthesize evidence on how GenAI nurtures creative learning across educational contexts; identifies patterns, effective practices, and implementation challenges	Secondary systematic review (contextual evidence; peer-reviewed book chapter; not a primary empirical study)	Studies across K-12 age groups (majority school-aged)	Formal and informal educational contexts	Multiple GenAI types (text-to-text, text-to-image, multi-modal)	Review and synthesis of GenAI integration across school subjects	Creative thinking, creative learning, cognitive engagement	Qualitative synthesis of included primary studies	Findings: GenAI supports creative learning through personalized content, reduced technical barriers, and increased engagement across school contexts; study types include LLM, image-gen, and multi-modal tools. Barriers: Wide variability in implementation quality; limited child-specific design; ethical and dependency concerns. Note: Included as secondary synthesis per expanded eligibility criteria (see Methods 2.1).
Han et al., 2023 USA (Han and Cai, 2023)	The implications of GenAI for visual storytelling among young learners	Qualitative user study (Think-aloud & Interviews)	10 children (ages 7–12)	Informal (Home or lab-based creative sessions)	Image generation (Midjourney, DALL-E 2) & Text (ChatGPT)	Visual storytelling tasks where children used GenAI to generate character and scene illustrations	Narrative creativity, Visual imagination, and Iterative design	Qualitative (Thematic analysis of interview transcripts and generated artifacts)	Findings: GenAI facilitates “visual scaffolding” for storytelling; children show high engagement in iterative prompting to refine visual details Barriers: Abstract AI concepts are difficult for children to grasp; inconsistency in AI-generated character styles; potential for “template-based” thinking
Lee and So, 2025 South Korea Lee and So, 2025 South Korea (Lee and So, 2025)	How students engage in the Creative Problem Solving (CPS) process using GenAI across social and scientific domains, and identify differences in prompting strategies between high and low performers	Mixed-methods (Case study)	Middle school students (Age 15)	Formal education/ Controlled environment (Solving PISA 2022 Creative Thinking problems)	Text-based (ChatGPT)	Students used ChatGPT as a partner/tool to “Generate Diverse Ideas,” “Generate Creative Ideas,” and “Evaluate/Improve Ideas” for PISA tasks	Divergent thinking (fluency and originality), evaluation and improvement of ideas, and domain-specific problem solving (social vs. scientific)	PISA 2022 Creative Thinking Rubrics (Appropriateness and Originality scores) and Prompt Analysis (Recursive vs. Linear prompting)	Findings: High-performing students used more “recursive” prompts (refining AI output), while low performers used “linear” prompts (accepting first answers). Students relied more on AI for social problems than scientific ones. Barriers: “Over-reliance” where students failed to justify AI-generated solutions; difficulty in evaluating the “originality” of AI suggestions
Fan et al., 2024 China (Fan et al., 2024)	The design space of an AI-empowered system (Story Prompt) for creative storytelling	Work in Progress (Qualitative: Focus groups & Preliminary evaluation)	12 elementary children (Grades 1–3 & 4–6) and 4 teachers	Formal education (Elementary school context)	Multi-modal: Text (LLM-based story generation) and Image (Stable Diffusion)	“Story Prompt” system: Children provide keywords/roles, AI generates plots and comics	Narrative creativity, Visual expression, and User engagement	Qualitative (Interviews, observation of AI-generated comics/stories)	Findings: Scaffolding is essential for different age groups (e.g., lower grades need more role-play); GenAI effectively transforms abstract ideas into visual comics Barriers: Children struggle with precise prompt engineering; potential for over-reliance on AI-generated plot templates
Horowitz-Kraus et al., 2025 Israel (Horowitz-Kraus et al., 2025)	To compare neural engagement and creativity levels in children during human-human vs. human-ChatGPT interaction	Experimental fMRI and behavioral study [bioRxiv preprint; not peer reviewed at search cut-off]	15 children (mean age 6.47 years) and 15 adults	Formal/ Laboratory (Neuroimaging center)	Text-based (ChatGPT via auditory/verbal interaction).	5-min semi-structured conversations on creative topics with AI vs. humans	Semantic creativity, Cognitive control, and Neural network modulation	Neuroimaging (fMRI BOLD signals) and behavioral semantic distance (SemDis)	Findings: Children showed significantly lower neural engagement in cognitive control and attention networks when interacting with ChatGPT compared to humans; lower behavioral creativity scores were also observed. Barriers: Over-reliance on AI may “bypass” necessary cognitive effort in children; reduced social/emotional cues in AI interaction
Kim et al., 2025 South Korea (Kim et al., 2025)	To evaluate the impact of a design-thinking-based AI program using GenAI on students' creative problem-solving	Quasi-experimental (Experimental vs. Control group)	117 middle school students (2nd year, approx. age 14)	Formal education (Middle school)	Text and Image (GenAI used for persona creation and storytelling)	10-session program; GenAI used in the “Empathize” and “Ideate” stages of design thinking	Creative problem-solving ability (Cognitive, Affective, and Motivating factors)	Quantitative (Standardized creative problem-solving scale; pre- and post-tests)	Findings: Significant improvement in creative problem-solving, especially in “Empathy” and “Idea Generation” through AI-assisted persona creation Barriers: Students need prior AI literacy to effectively prompt the system; time constraints in formal curricula
Aldina et al., 2025 Indonesia (Aldina et al., 2025)	The impact of GenAI-integrated digital storytelling on middle schoolers' creativity and engagement	Quasi-experimental (Pre-test/Post-test design)	120 middle school students (divided into experimental and control groups)	Formal education (Middle school)	Multi-modal: Text (ChatGPT for scripts) and Image (Midjourney/ DALL-E for visual assets)	Digital storytelling projects: Experimental group used GenAI for assets; control group used traditional digital tools	Narrative originality, visual complexity, and student engagement	Quantitative (Creativity assessment rubrics and engagement surveys)	Findings: Experimental group showed significantly higher scores in narrative originality and visual assets; GenAI reduced the “technical barrier” to expression Barriers: Risk of “creative laziness” (over-reliance on AI suggestions); ethical concerns regarding the authenticity of student work
Chin et al., 2024 USA (Chin et al., 2024)	To compare children's creative storytelling styles when interacting with ChatGPT vs. a parent	Qualitative user study (Comparative interaction analysis)	10 children (ages 4–9) and their parents	Informal (Home-based storytelling sessions)	Text-based (ChatGPT via voice-to-text interface)	Collaborative storytelling: Child starts a story, then takes turns with ChatGPT or a parent to complete it	Narrative coherence, vocabulary diversity, and turn-taking engagement	Qualitative (Thematic analysis of transcripts; comparison of interactive scaffolding styles)	Findings: ChatGPT provides more “elaborative” questions that spark new ideas; parents focus more on “scaffolding” logic and coherence Barriers: ChatGPT sometimes produces overly long or complex responses for younger children; lacks the emotional attunement of a parent
Fan et al., 2025 China (Fan et al., 2025)	To design and evaluate “Story Prompt,” a multi-modal GenAI system for children's creative storytelling	Mixed-methods: Comparative user study (System vs. Traditional) and interviews	24 elementary school children (Grades 1–6)	Formal education (Elementary school)	Multi-modal: LLM (Plot generation) and Stable Diffusion (Comic/Image generation)	60-min storytelling sessions comparing GenAI-assisted comic creation vs. manual drawing/writing	Narrative complexity, Visual creativity, and Self-efficacy in creation	Narrative scoring rubrics, creativity self-report scales, and semi-structured interviews	Findings: GenAI significantly reduces “blank page syndrome” and lowers the barrier for visual expression; children shifted from “execution” to “curation.” Barriers: Prompt-dependence (lower grades rely more on system defaults); “Image-to-Story” consistency issues
Panggabean et al., 2026 Indonesia (Panggabean et al., 2026)	To examine the effectiveness of ChatGPT-4 integrated with Contextual Teaching and Learning (CTL) in optimizing poetry writing and creative thinking	Quantitative (Quasi-experimental, Non-Equivalent Control Group Design)	Elementary school students (Experimental vs. Control groups)	Formal education (Elementary school)	Text-based (ChatGPT-4)	Integration of ChatGPT-4 within the CTL framework to assist students in finding metaphors, vocabulary, and structural inspiration for poetry	Creative thinking skills (Fluency, Flexibility, Originality) and poetry writing proficiency	Quantitative (Pre-test/Post-test; N-Gain score analysis via JAMOVI/JASP)	Findings: Highly significant improvement in all creativity indicators; ChatGPT-4 served as an effective bridge between abstract concepts and creative expression. Barriers: Requires strong teacher facilitation to ensure students don't lose their personal “voice” or emotional depth in poetry
Pebriana, 2025 Indonesia (Pebriana, 2025)	The effect of GenAI as digital scaffolding on textual quality and narrative writing creativity in elementary L1 learning	Quasi-experimental (Pretest-posttest control group design)	58 upper-grade elementary students (approx. ages 10–12)	Formal education (Elementary school, Indonesian language class)	Text-based (GenAI assistant)	Digital scaffolding across three stages: planning (idea generation), drafting (coherence), and revising (narrative refinement)	Narrative creativity (Originality, plot development, and imaginative elements) and textual quality (coherence/ cohesion)	Quantitative (Rubrics based on Torrance Tests of Creative Thinking and linguistic markers)	Findings: Significant improvement in both textual quality and narrative originality; AI was more effective in the “planning” stage than in “drafting.” Barriers: Students sometimes accepted AI suggestions without critical reflection; limited impact on students with very low initial literacy
Picton and Clark, 2024 UK (Picton and Clark, 2024)	To explore children and young people's engagement with, attitudes toward, and use of GenAI for literacy and creative activities	Large-scale National Survey (Annual Literacy Survey)	15,379 students	Mixed (Formal schooling and informal/ personal use).	Text-based (ChatGPT, Gemini, Claude) and Image-based (DALL-E, etc.)	Self-reported frequency and nature of AI use (e.g., generating story ideas, refining drafts, or creative brainstorming)	Narrative ideation (brainstorming), creative writing confidence, and artistic exploration	Quantitative (Survey statistics) and Qualitative (Open-ended participant reflections)	Findings: 3 in 4 students have used GenAI; key creative uses include brainstorming story ideas and checking drafts; AI boosts confidence for struggling writers Barriers: Concerns over “losing their own voice”; fear of AI replacing human creativity; gap in school guidance on ethical use
Silvennoinen et al., 2024 Finland (Silvennoinen et al., 2024)	How children use T2I GenAI to co-create and visualize solutions for sustainability	Qualitative participatory design study [SSRN working paper; not peer reviewed at search cut-off]	12 children (ages 10–12)	Formal education (Primary school STEAM workshop)	Image-based (Text-to-Image AI, e.g., Midjourney/ DALL-E)	90-minute workshop: Children ideated sustainability themes and used AI to generate visual solutions	Visual imagination, problem-solving, and thematic originality in sustainability	Qualitative (Analysis of prompts, generated images, and child-led descriptions)	Findings: GenAI facilitates “visual prototyping” of complex ideas; children used AI to bridge the gap between abstract concepts and concrete visuals Barriers: Technical limitations in rendering specific details; ethical/environmental concerns about AI itself were raised by children
Kiziltaş, 2025 Türkiye (Kiziltaş, 2025)	The effect of ChatGPT's feedback mechanism on the creative writing skills and writing self-efficacy of primary school students	Quantitative (Quasi-experimental pre-test/post-test control group design)	62 fourth-grade students (Age 10; 31 in experimental, 31 in control)	Formal education (Primary school classroom)	Text-based (ChatGPT)	Students used ChatGPT under teacher guidance to receive real-time feedback, suggestions, and corrections on their creative writing drafts	Creative writing quality (originality, fluency, flexibility) and Writing Self-Efficacy (confidence in writing tasks)	Creative Writing Product Evaluation Scale; Creative Writing Self-Efficacy Scale	Findings: The experimental group showed significantly higher scores in both creative writing products and self-efficacy compared to the control group. AI feedback served as an “on-demand tutor” that reduced writing anxiety Barriers: The need for strict teacher supervision to ensure AI suggestions are used for learning rather than simple copying
Luo and Tahir, 2025 UK (Luo and Tahir, 2025)	The potential of ChatGPT in enhancing STEAM arts lesson planning for fostering 21st-century 4C skills	Mixed-methods (Experimental study, questionnaires, and interviews)	13 teachers and 6 professors (evaluating plans for school-aged children)	Formal education (STEAM Arts Education)	Text-based (ChatGPT)	Comparative study: Traditional lesson planning vs. ChatGPT-assisted planning using a specific prompt framework	Instructional creativity, interdisciplinary integration, and 4C skills cultivation	Quantitative (Rubric scores: 17.0 traditional vs. 21.0 AI-assisted) and Qualitative (Thematic analysis)	Findings: ChatGPT significantly improves the quality and efficiency of creative lesson plans; supports interdisciplinary “STEAM” approachesBarriers: Concerns about the pedagogical depth of AI suggestions; potential for generic/repetitive artistic activities if not guided by expert prompts
Awanda and Pangestu, 2025 Indonesia (Awanda and Pangestu, 2025)	The role of ChatGPT in assisting elementary students with short story writing while balancing creativity and dependence	Qualitative (Phenomenology); In-depth interviews and observations	Elementary school teachers (as primary informants) and students	Formal education (Elementary schools)	Text-based (ChatGPT)	Used as an AI writing instructor to provide feedback, suggestions, and help organize thoughts during story composition	Narrative articulation, writing confidence, and critical thinking	Qualitative (Thematic analysis of interview transcripts and observational data)	Findings: ChatGPT fosters a more inclusive learning environment; boosts students' confidence and “distinctive voices”; improves grammar and syntax understanding Barriers: The risk of student “dependence” on AI; ethical concerns regarding original authorship
Wang et al., 2025 China (Wang et al., 2025)	The usage and impact of GenAI on elementary students' collaboration and creativity in offline group project-based art courses	Qualitative (Case study: Observations and focus group interviews)	18 elementary school students (aged 9–11)	Formal/Semi-formal (Offline Art Course, Group-based projects)	Multi-modal (Text-to-Image tools like Midjourney/ DALL-E)	GenAI used for “Idea Sketching” and “Visual Reference” during group discussions to design creative art installations/projects	Group creativity, collaborative ideation, and visual aesthetic diversity	Qualitative (Thematic analysis of group interaction records and final art projects)	Findings: GenAI facilitates “shared mental models” among students; it helps groups quickly visualize and align divergent ideas Barriers: Technical hurdles in prompt precision; potential “AI-driven conformity” where groups might follow AI suggestions too closely without critique
Yang et al., 2025 Hong Kong, China (Yang et al., 2025)	To conceptualize and evaluate a “Creative Project Approach” that integrates GenAI agents and robotics to foster creativity in early childhood education (ECE)	Qualitative & Framework Development (Pedagogical conceptualization and case application)	Young children (Early Childhood/Preschool age)	Formal education (Early Childhood Education settings)	AI Agents and Robotics	A 5-step pedagogical framework: (1) Identifying needs, (2) Child-robot interaction, (3) Creative ideation with AI, (4) Collaborative execution, (5) Reflection/Evaluation	Creative learning, problem-solving in digital contexts, and tech-supported ideation	Qualitative (Teacher observations, child engagement patterns, and pedagogical framework validation)	Findings: AI agents act as “creative partners” that provide personalized scaffolding; the hybrid use of AI and physical robots enhances embodied learning Barriers: Lack of teacher guidance in tailoring AI agents for specific ECE tasks; ethical concerns regarding screen time and data privacy for minors
Pade and Monga, 2025 India (Pade and Monga, 2025)	To explore primary school students' spatial design preferences for their learning environments using GenAI as a co-design tool	Qualitative & Participatory Design (Workshops using Text-to-Image AI)	Primary school students (Ages 7–11)	Formal education (Primary school classrooms)	Image-based (Text-to-Image Gen AI, Midjourney/ DALL-E)	Children provided descriptive prompts of their “ideal classroom” to the AI, which then generated 3D-like spatial visualizations for discussion	Spatial imagination, environmental agency, and visual articulation of complex abstract needs	Qualitative (Thematic analysis of AI-generated images, children's verbal explanations, and design patterns)	Findings: GenAI empowers children to act as “mini-architects,” bypassing their lack of technical drawing skills; children prioritize biophilic elements and flexible/movable furniture Barriers: Abstract concepts sometimes resulted in “hallucinations” in AI-generated architecture; children's prompts were initially limited by their existing classroom experiences
Shrivastava et al., 2024 India (Shrivastava et al., 2024)	How young children (pre-literate/early-literate) interact with and perceive ChatGPT during open-ended, child-led exploration.	Qualitative (*In-situ* observations and co-exploration sessions)	12 children (ages 5–8) from two different cultural contexts	Informal/Home-based (Family environments)	Text-based (ChatGPT via voice-to-text interface)	Open-ended interaction: Children were encouraged to “talk” to the AI about anything, from weather and stories to personal feelings	Imaginative curiosity, conversational engagement, and anthropomorphic perception (viewing AI as a peer)	Qualitative (Thematic analysis of interaction transcripts and behavioral observations)	Findings: Children treat ChatGPT as a “knowledgeable peer”; cultural background influences the types of questions (e.g., pragmatic vs. imaginative). AI sparks curiosity by providing immediate, albeit sometimes complex, answers Barriers: The “black box” nature of AI—children struggle to understand where the info comes from; voice-to-text errors cause frustration; AI's inability to understand child-specific emotional nuances
Hu X. et al., 2025 Hong Kong China (Hu Z. et al., 2025)	To design and evaluate “MusicScaffold,” a GenAI system that bridges machine efficiency and human cognitive/affective growth in music education	Mixed-methods prototype evaluation [arXiv preprint; not peer reviewed at search cut-off]	Adolescents (Ages 12–18)	Formal/Non-formal education (Creative Music Education)	Audio/Music-based (Generative AI for symbolic music and audio generation)	An AI coaching system that provides symbolic explanations and prompts reflection, acting as a “co-creation partner” rather than a mere generator	Music composition self-efficacy, cognitive growth (understanding music theory), and affective growth (creative identity)	Quantitative (Self-report scales on growth and efficiency) and Qualitative (Interview thematic analysis)	Findings: GenAI significantly lowers the barrier to music production; “Symbolic Explanations” help adolescents learn underlying patterns rather than just consuming AI output Barriers: Over-reliance on AI “quick fixes” can hinder deep skill acquisition; need for Al to “coach” by prompting reflection instead of just executing

The evidence set comprises 22 primary empirical studies and two secondary syntheses (Ali et al., 2024; Pérez-Jorge et al., 2026), which are labeled and used only for contextual interpretation. Publication status is also indicated for non-peer-reviewed sources, including the Horowitz-Kraus et al. (2025) bioRxiv preprint. Asterisks identify other boundary cases justified under Section 2.1 (e.g., age-range or teacher-mediated designs). Such sources should not be interpreted as equivalent in evidentiary weight to peer-reviewed primary empirical studies.

**Figure 1:**
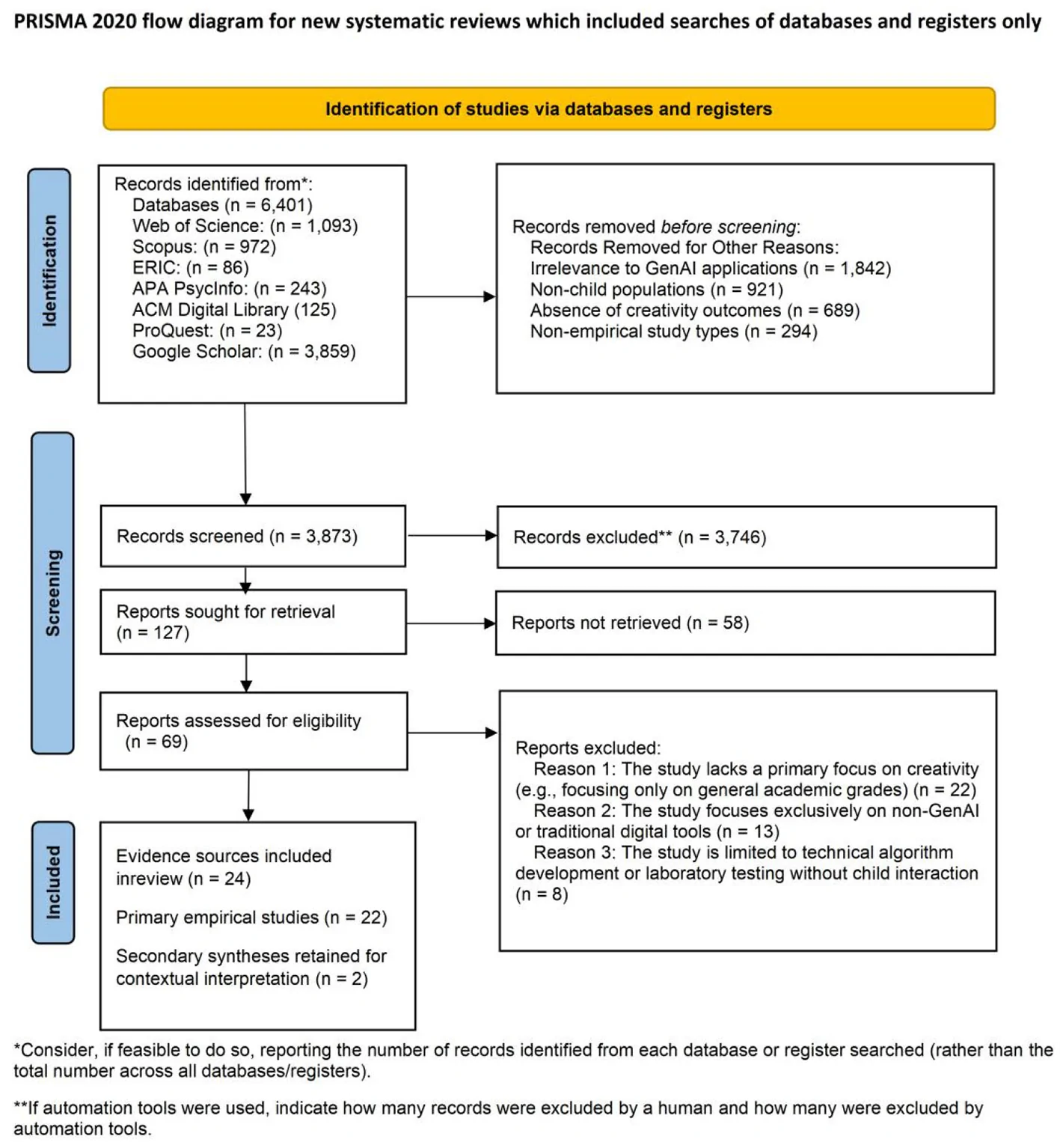
PRISMA flowchart.

### 3.2 Temporal and geographical characteristics of published studies

The 24 included evidence sources were published between 2023 and the May 2026 search update: one in 2023, seven in 2024, fourteen in 2025, and two in 2026 (Figure 2). This distribution indicates rapidly increasing scholarly attention, but it should not be interpreted as maturation or increasing rigor of the field. It includes primary studies, two secondary syntheses, and non-peer-reviewed outputs. The concentration in 2025 may reflect the wider availability of GenAI tools and growing educational interest; causal explanations for the publication trend were not tested.

**Figure 2:**
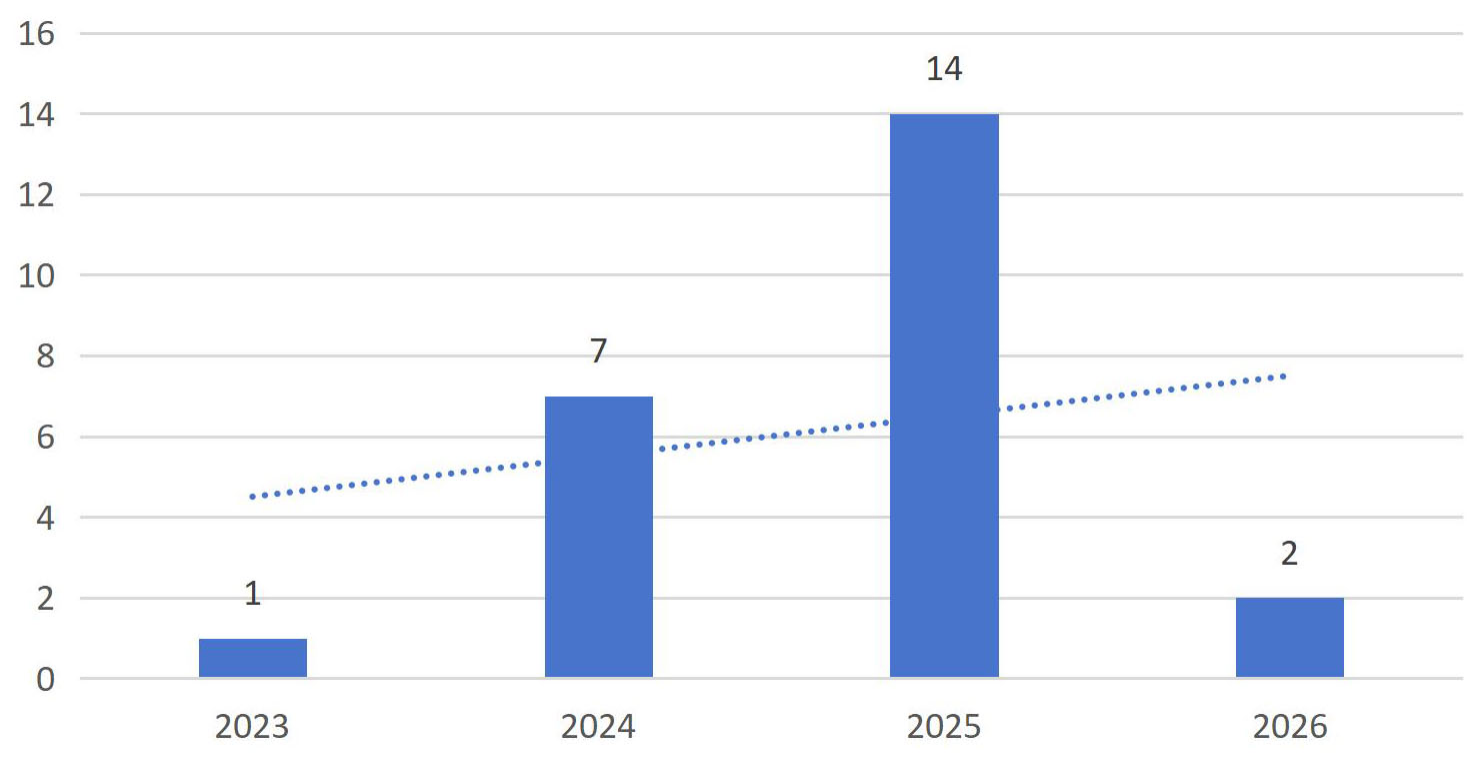
Annual publication count of included studies.

Geographically Figure 3, research in this field is concentrated in Southeast Asia, East Asia, and North America, with scattered contributions from Europe, West Asia, and South Asia, reflecting regional disparities in GenAI research investment, educational technology infrastructure, and national priorities for creative skill development. Indonesia leads with 5 studies, driven by its national agenda to integrate digital technologies into primary and secondary curricula, prioritize creative education as a driver of social innovation, and leverage GenAI to expand access to high-quality creative mentor ship across urban and semi-urban communities. China, including Hong Kong, with five studies, focusing on multi-modal GenAI system design, classroom-based creative interventions, and collaborative creative learning, fueled by significant government investment in AI education research and a robust ecosystem of EdTech startups developing child-centric tools. The USA contributes three studies, with a distinct focus on qualitative user experience research, neurocognitive analyses of child-AI interaction, and comparative studies of AI vs. human scaffolding in creative activities, reflecting a research culture that emphasizes individual learner experiences and cognitive developmental outcomes. South Korea accounts for two studies, centered on GenAI-supported creative problem-solving and the analysis of student prompting strategies, aligning with its longstanding focus on integrating technology into STEM/STEAM education and fostering global competitiveness in creative industries. The UK contributes two studies, focusing on large-scale national surveys of GenAI use among children and STEAM arts lesson planning, reflecting a policy emphasis on evidence-based EdTech adoption and equitable access to creative learning resources. India also contributes two studies, exploring spatial design preferences and early childhood AI interaction, reflecting growing interest in adapting global GenAI practices to local educational contexts and addressing creative skill gaps in diverse learner populations. Finland, Spain, Turkey, Bangladesh, and Israel each contribute one study, with European research emphasizing student-centered design and sustainability-focused creative learning, and West Asian research (Israel) pioneering neuroimaging-based analyses of GenAI's cognitive impacts. This geographical concentration stems from three key factors: higher levels of public and private investment in AI and educational technology in technologically advanced regions, explicit national curricular policies prioritizing creativity as a core competency, and the presence of interdisciplinary research networks that bridge computer science, education, and developmental psychology, enabling rigorous exploration of GenAI's role in children's creative development.

**Figure 3:**
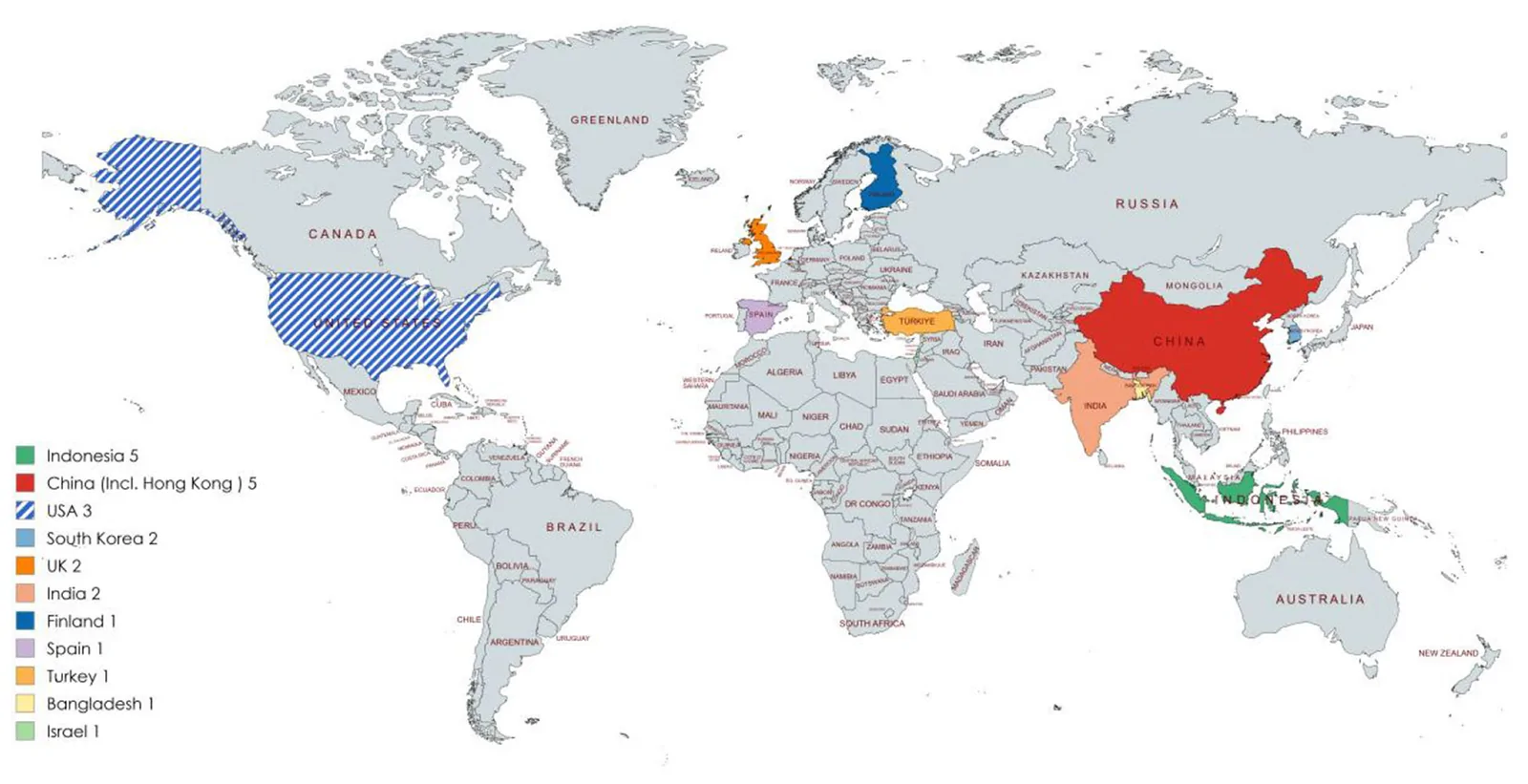
Geographical distribution of included studies.

### 3.3 Research aims

Research aims were coded inductively using a two-stage process. The 22 primary empirical studies were grouped into four descriptive clusters: tool/system design and evaluation; reported creative or cognitive outcomes; child-AI interaction and user experience; and contextual applications. The two secondary syntheses were charted separately and used only to contextualize implementation and ethical themes. Because several studies addressed overlapping aims, the clusters map emphases rather than mutually exclusive or comparative-effectiveness categories. References to equity or expanded access in the source literature are reported as proposed aims or interpretations, not as empirically established outcomes.

### 3.4 Research design and measurement instruments

Research designs were classified from the reported primary method. Among the 22 primary empirical studies, designs included qualitative/participatory studies, quantitative surveys or quasi-experiments, and mixed-methods evaluations; the two literature reviews were classified separately as secondary syntheses. This distinction corrects the earlier presentation in which all 24 sources were assigned to empirical design categories. The evidence base remains dominated by small, short-term, exploratory studies, with few controlled comparisons and no longitudinal designs.

Measurement approaches varied widely and included artifact analysis, interviews, observations, prompt logs, self-report scales, creativity rubrics, pre/post measures, and one preliminary neuroimaging study. This heterogeneity prevented meaningful pooling. Only a minority used standardized measures or comparison groups, and reported statistical significance was not treated as equivalent to established efficacy. The two secondary syntheses did not contribute participant-level measurements.

### 3.5 Target cohort and research setting

The primary studies mostly involved school-aged children, but the corpus also contains age-boundary cases, teacher-mediated designs, and two secondary syntheses without a single child cohort. Upper-elementary and middle-school learners were most frequently represented. Younger children often participated through multimodal or adult-facilitated interfaces, whereas older learners more often used text-based tools. These descriptive patterns do not establish age-specific effectiveness, and the broad 5–18-year span limits developmental generalization.

Research settings are clearly split between formal educational environments (16 studies) and informal learning contexts (eight studies), reflecting two distinct application paradigms of GenAI for children's creative thinking. Formal settings include primary and middle school classrooms, school ICT labs, STEAM workshops, and university neuroimaging laboratories, with GenAI interventions integrated into core academic subjects, e.g., science, language arts, visual art, or dedicated creative education programs. Informal settings encompass home-based learning, after-school design workshops, lab-based child-led creative sessions, and family storytelling activities prioritizing child autonomy, self-directed exploration, and creative interest. In these contexts, GenAI is often used as a flexible tool for personal creative expression, e.g., generating visual art, writing stories, or collaborative creative projects with peers or family members.

### 3.6 GenAI types and modalities

Text-based and multi-modal GenAI are the dominant modalities used in the included studies, with image-based GenAI as a secondary focus, and audio/music-based GenAI minimally represented, reflecting the current maturity, accessibility, and educational relevance of different GenAI types for K-12 creative learning. Text-based GenAI is the most widely used modality (12 studies), primarily comprising LLMs such as ChatGPT 3.5/4, Gemini, and Claude. Text-based GenAI is applied across a range of creative domains, including creative writing (short stories, poetry), story plotting, creative problem-solving, and lesson planning support for teachers facilitating creative activities. Multi-modal GenAI (seven studies), integrating text and image generation, follows closely as the second most used modality. This modality addresses both the narrative and visual dimensions of children's creativity, enabling the translation of abstract textual ideas into concrete visual outputs, e.g., comic strips, scene illustrations, and character designs. Multi-modal GenAI is particularly valuable for creative storytelling, digital art projects, and collaborative design activities, allowing children to express complex creative ideas that combine words and images. Image-based GenAI (four studies), consisting of text-to-image tools such as Midjourney and DALL-E 2, is used primarily for visual creative expression, including visual storytelling, sustainability-focused design visualization, and student-led spatial design of learning environments. Its key value lies in decoupling creative intent from technical artistic execution, enabling children with limited fine motor skills to visualize their creative ideas with precision, thereby reducing creative anxiety and expanding participation in visual creative activities. Audio/music-based GenAI (one study) is the least represented modality, with only one study exploring a GenAI music coaching system for adolescent music composition and education. This modality supports music theory learning, melody generation, and creative expression through sound, though its limited representation reflects the relative immaturity of user-friendly, child-appropriate audio GenAI tools compared to text and image modalities.

### 3.7 Intervention forms and durations

GenAI interventions in the included studies are characterized by short-term, structured activities and moderate-term program-based designs, with no long-term longitudinal interventions. Workshop-based interventions (nine studies) are the most common form across both formal and informal settings, typically consisting of 90-min sessions with 4–10 total sessions delivered over 1–4 months. A defining feature of workshop-based interventions is their participatory nature: children often co-create AI prompts, iterate on AI-generated outputs, and collaborate with peers or facilitators to refine their creative work. This format allows for deep engagement with GenAI tools while maintaining flexibility to adapt to children's interests and needs. Classroom-based integrated interventions (seven studies) are embedded directly into formal school curricula, ranging from single 60-min standalone sessions to 10-week sustained programs. These interventions are aligned with curricular objectives and delivered by classroom teachers, focused on scalable GenAI integration into routine instruction. Examples include AI-enhanced game-based learning for scientific creativity, digital storytelling projects for language arts, and poetry writing with ChatGPT integrated into CTL frameworks. Comparative interaction interventions (5 studies) involve children completing identical creative tasks with two different forms of scaffolding, typically GenAI vs. human support or different GenAI modalities. These interventions have short durations (5–60 min per task) and focus on analyzing interaction dynamics, prompting strategies, and comparative effectiveness in supporting creative outcomes. System design and evaluation interventions (three studies) focus on developing and testing custom, child-centered GenAI systems for creative learning. Intervention durations vary based on the system development stage, from preliminary user evaluations (1–2 sessions) to full-scale user studies (12 weeks), and prioritize assessing usability, acceptability, and alignment with children's developmental needs. A universal characteristic of all included interventions is that they are facilitated (by teachers, researchers, or parents), with no studies exploring unguided, child-led GenAI use for creative thinking. Additionally, all interventions are short to moderate in duration ( ≤ 4 months), with no longitudinal studies (≥6 months) assessing the long-term impacts of sustained GenAI use on children's creative thinking development, highlighting a critical gap in understanding how GenAI influences creative skill retention and developmental trajectories over time.

### 3.8 Targeted creativity dimensions

The included studies target a diverse range of creativity dimensions, with a primary focus on divergent thinking and narrative creativity, and secondary emphasis on creative self-efficacy and domain-specific creative problem-solving. Divergent thinking is the most frequently targeted dimension (11 studies, with explicit focus on its three foundational sub-components: fluency (the quantity of ideas generated), flexibility (the variety of idea categories), and originality (the uniqueness of ideas relative to peers or norms). This dominant focus reflects divergent thinking's central role in early and middle childhood creative development, serving as a precursor to more complex creative skills, and GenAI's inherent strength in scaffolding idea generation to address the common cognitive barrier of “idea scarcity.” Narrative creativity (nine studies), encompassing story plot development, visual storytelling, narrative coherence, vocabulary diversity, and character creation is the second most targeted dimension. Narrative creativity interventions often aim to support children in translating abstract ideas into structured, engaging narratives with GenAI providing scaffolding such as plot prompts, character suggestions, or visual illustrations.

Creative self-efficacy (five studies), the subjective belief in one's ability to generate creative ideas and produce creative work is a key affective dimension targeted in the studies. Research consistently demonstrates that GenAI can boost creative self-efficacy by reducing technical barriers to creative expression, and providing immediate, positive feedback on creative ideas, helping children build confidence in their creative abilities and fostering a willingness to engage in future creative challenges. Domain-specific creative thinking (seven studies) is targeted across a range of academic and creative domains, including scientific creativity, visual art creativity, music composition creativity, sustainability-focused creative problem-solving, and social/scientific creative problem-solving.

Other targeted creativity dimensions include collaborative creativity (three studies), exploring how GenAI supports group creative ideation, shared mental models, and collaborative refinement of ideas, and elaboration (four studies), the ability to develop, detail, and refine creative ideas. Elaboration is a critical sub-component of divergent thinking that is surprisingly underrepresented, despite GenAI's potential to support iterative idea refinement through recursive prompting and feedback. Additional niche dimensions include spatial imagination (two studies), focused on visualizing spatial relationships and designing physical environments, and semantic creativity (one study), exploring the generation of novel semantic connections and ideas.

## 4 Discussion

### 4.1 Synthesis of core findings

This systematic scoping review mapped 24 evidence sources published between 2023 and 2026: 22 primary empirical studies and two secondary syntheses used for context. Research output increased rapidly and was concentrated in Southeast Asia, East Asia, and North America. The primary studies used heterogeneous qualitative, quantitative, and mixed-methods designs, commonly emphasizing user experience and short-term intervention outcomes; longitudinal, independently replicated, and neurocognitive evidence remained scarce. These patterns describe the available literature but do not establish comparative effectiveness.

Across the primary studies, GenAI was usually facilitated by adults and delivered through text-based LLMs or multimodal text/image tools. Several studies reported reduced technical barriers, stronger creative self-efficacy, or improvements in task-specific creativity measures. These findings should be read as reported outcomes rather than confirmed efficacy because no meta-analysis, effect-size synthesis, or formal risk-of-bias appraisal was conducted. Positive-result and publication bias are plausible in this new field. Moreover, the bioRxiv preprint by Horowitz-Kraus et al. (2025), which has not undergone peer review, reported lower neural engagement in cognitive-control and attention networks and lower behavioral creativity during ChatGPT interaction than during human interaction. It is therefore treated as preliminary and hypothesis-generating, not as decisive neuroimaging evidence. The contrast between positive task-performance reports and this preliminary result highlights the need to distinguish AI-assisted performance from durable creative development. The near-absence of rural and underserved samples also means that democratization and equity claims are currently unsubstantiated.

Cross-study comparison further reveals meaningful differential effectiveness across age groups and GenAI modalities that the original literature largely fails to synthesize. For younger children, multi-modal and embodied tools, combining text-to-image generation with physical or robotic elements (Chin et al., 2024; Shrivastava et al., 2024; Yang et al., 2025), appear better suited to developmental needs, as these children lack the precise linguistic and metacognitive skills required for effective text-based prompting but can engage creatively through visual and tactile affordances. By contrast, text-based LLMs show stronger and more consistent outcomes among older children and young adolescents, who possess sufficient language command to craft iterative prompts and critically evaluate AI-generated outputs (Kim et al., 2025; Kiziltaş, 2025; Lee and So, 2025; Panggabean et al., 2026). This developmental gradient has significant implications for tool design and pedagogical sequencing: a GenAI integration approach appropriate for middle school students may actively frustrate younger learners, yet the reviewed literature contains no study that systematically examines developmental readiness thresholds for different GenAI modalities. From a theoretical standpoint, these patterns align with Vygotsky's Zone of Proximal Development (Fani and Ghaemi, 2011), insofar as GenAI's scaffolding value is contingent on the fit between a child's current developmental capabilities and the cognitive demands of the AI interface. When this fit is appropriate, GenAI functions as a genuine proximal scaffold, extending creative performance beyond what the child could achieve independently; when it is mismatched, as may occur with complex text interfaces for young children or over-simplified tools for adolescents, it risks either overwhelming the learner or bypassing the effortful cognitive engagement that drives genuine skill development (Csikszentmihalyi, 1997; Vygotsky and Cole, 1978), componential model of creativity further contextualizes these findings: the domain knowledge, creative process, and field evaluation components all require developmentally calibrated support that neither a uniform GenAI tool nor a single pedagogical approach can provide across the full 5–18 age range represented in this review. Advancing the field, therefore, requires both age-stratified empirical research and theoretically grounded tool design that treats developmental appropriateness not as a secondary consideration but as a primary design constraint.

### 4.2 The integration of GenAI into children's creative thinking development

The included literature suggests three possible roles for GenAI in children's creative activity: scaffold, co-creation partner, and creative amplifier across idea generation, expression, and refinement. These are descriptive roles reported in the literature, not established causal mechanisms. They may reduce some technical barriers for learners who can access suitable devices, connectivity, adult facilitation, and age-appropriate interfaces; they cannot be assumed to remedy structural inequalities in staffing, infrastructure, language, disability access, or digital literacy.

At the idea-expression stage, several studies reported that text-to-image and text-based tools helped children translate verbal concepts into visual or written outputs (Fan et al., 2025; Fitriyani et al., 2025). This may broaden participation for some learners with limited drawing or writing proficiency. However, the reviewed samples were largely drawn from urban or technologically supported settings, so the evidence does not show that these tools reduce population-level inequity or benefit children who lack reliable access, local-language support, or trained facilitation.

At the refinement and collaboration stage, some studies described recursive prompting, rapid prototyping, and shared visual references (Silvennoinen et al., 2024; Wang et al., 2025). The evidence also indicates risks of prompt dependence, reduced cognitive effort, and loss of personal voice. Human facilitation therefore remains important, and any claims about executive-function development require direct longitudinal measurement. Overall, GenAI should be framed as a conditional supplementary resource whose value depends on developmental fit, access, supervision, and task design, rather than as an inherently inclusive or equitable solution.

### 4.3 Implementation barriers and dilemmas for educators

Educators face a multifaceted set of implementation barriers and dilemmas when integrating GenAI into creative education for children, spanning technical, pedagogical, systemic, and attitudinal challenges that limit scalable, sustainable use in formal classroom settings (Aldina et al., 2025; Kim et al., 2025). A primary and pervasive technical barrier is the educator AI literacy deficit: the majority of K-12 teachers lack foundational knowledge and practical skills to effectively use GenAI tools, design age-appropriate creative interventions, and teach children critical prompt engineering strategies skills essential for maximizing GenAI's scaffolding potential. This literacy gap is compounded by the rapid evolution of GenAI tools: new models, features, and capabilities are released regularly, requiring ongoing professional development that most educational institutions lack the time, funding, or resources to provide (Luo and Tahir, 2025). Additional technical barriers include inadequate school infrastructure unstable internet connectivity, limited device access, and restrictive internet policies that block popular GenAI tools challenges acutely felt in low-resource setting “digital divide” among educators, with only a small subset of technically proficient, forward-thinking teachers able to integrate GenAI into creative education, while the majority remain excluded.

Pedagogically, educators face the fundamental dilemma of balancing AI scaffolding and child creative autonomy: over-scaffolding with GenAI risks fostering over-reliance on AI for idea generation and expression, bypassing the cognitive effort required for independent creative thinking and leading to declines in children's ability to generate original ideas autonomously (Kiziltaş, 2025; Lee and So, 2025). Conversely, under-scaffolding leaves children unable to effectively use GenAI due to limited prompt engineering skills, leading to frustration, disengagement, and failure to realize GenAI's creative potential. This dilemma is amplified by the lack of evidence-based pedagogical frameworks for GenAI integration: most current interventions are *ad-hoc* and not aligned with developmental psychology principles for children's creative thinking, leaving educators without clear guidance on structuring AI-augmented activities, sequencing scaffolding, or balancing AI and human support. Educators also face significant classroom management and time constraints: GenAI interventions require flexible lesson planning, individualized support for prompt engineering, and time for iterative creative exploration, all conflicting with rigid curricular timelines, standardized testing pressures, and large class sizes common in K-12 education worldwide (Kim et al., 2025). A further pedagogical challenge is the assessment of AI-augmented creative work: traditional creativity rubrics do not account for AI's role in the creative process, leaving educators uncertain how to evaluate children's original contributions, distinguish between AI-generated and child-generated ideas, and avoid penalizing students who use AI effectively as a creative tool (Awanda and Pangestu, 2025; Picton and Clark, 2024).

Systemic and attitudinal barriers further hinder implementation: the lack of clear school and national policies for GenAI use in K-12 creative education leaves educators without direction on ethical use, data privacy, academic integrity, and acceptable AI use in creative work (Picton and Clark, 2024). This policy vacuum creates ambiguity and risk aversion, with educators fearing disciplinary action or parental criticism for inappropriate GenAI use. Additionally, educators face parental and institutional skepticism: many parents and administrators fear AI will replace human creativity, reduce children's cognitive effort, and erode traditional skills, e.g., drawing, writing, and critical thinking, resistance that persists even when educators recognize GenAI's potential for equity and creative growth (Horowitz-Kraus et al., 2025; Picton and Clark, 2024). Collectively, these technical, pedagogical, systemic, and attitudinal barriers mean GenAI integration in children's creative education is currently limited to progressive, well-resourced schools and research settings with minimal scalable, sustainable implementation in mainstream K-12 education. Addressing these barriers requires coordinated action across stakeholders to build educator capacity, develop evidence-based frameworks, and create supportive policy environments.

### 4.4 Ethical and safety issues in GenAI for children's creative thinking

The use of GenAI to foster children's creative thinking raises unique ethical and safety issues rooted in children's inherent vulnerabilities, limited critical thinking, underdeveloped metacognition, right to privacy, and GenAI's core characteristics (algorithmic bias, black box functionality, data collection practices) issues amplified by the lack of child-centered GenAI design and regulatory frameworks tailored to K-12 education (Ali et al., 2024; Shrivastava et al., 2024).

A critical safety and ethical concern is children's data privacy and security: mainstream GenAI tools often collect and store large volumes of children's data, including creative prompts, AI-generated outputs, interaction history, and even voice/image data, with many failing to comply with international child data protection regulations (Picton and Clark, 2024; Yang et al., 2025). These tools lack age-appropriate data collection, storage, and deletion practices, putting children's sensitive personal and creative data at risk of breaches, misuse, or commercial exploitation. Children's creative prompts and outputs often reveal their interests, thoughts, feelings, and developmental levels. Unauthorized collection and use of this data violates their fundamental right to privacy and can have long-term consequences for their digital identity. Another major safety issue is the black box nature of GenAI and its potential to generate harmful, inappropriate, or inaccurate content: children lack the critical thinking skills to understand how GenAI generates outputs, leading to uncritical acceptance of AI-generated ideas, and tools may produce violent, sexual, discriminatory, or factually inaccurate content in response to ambiguous prompts (Newman et al., 2024; Shrivastava et al., 2024). This risk is acute for young children, who cannot distinguish between appropriate/inappropriate content or fact/fiction in AI outputs, exposing them to harm and distorting their understanding of creative expression.

A central ethical concern for creative development is the risk of cognitive dependency and reduced critical thinking: over-reliance on GenAI for idea generation can bypass the effortful cognitive engagement required for independent creative thinking, leading to declines in children's ability to generate original ideas, evaluate outputs, and engage in iterative refinement (Horowitz-Kraus et al., 2025; Lee and So, 2025). This dependency is particularly problematic for children in the 6–15-year-old developmental window, whose executive functions and creative skills are still maturing; they need ample unguided creative exploration to build these capabilities. Another key ethical issue is creative ownership and academic integrity: the blurring of lines between children's input and AI-generated output raises complex questions about who owns creative work produced with GenAI, how to teach children about attribution, and how to maintain integrity in creative education (Awanda and Pangestu, 2025; Newman et al., 2024). Children may struggle to distinguish between using AI as a tool and plagiarizing AI content without clear guidance; they may develop poor creative and academic habits that persist into adolescence and adulthood. Collectively, these ethical and safety issues highlight the urgent need for child-centered GenAI design, including bias mitigation, robust privacy protection, age-appropriate content filtering, simplified prompt interfaces, and integrated digital literacy education to teach children to use GenAI critically, ethically, and autonomously.

### 4.5 Recommendations for stakeholders

Given the limited and uneven evidence base, the following recommendations are precautionary implementation principles rather than endorsements of broad adoption. Educators considering GenAI should begin with small, supervised, age-appropriate pilots; preserve non-AI routes to participation; document when and how AI contributes to the creative process; and monitor independent creativity, not only AI-assisted products. AI literacy, critical evaluation, authorship, privacy, and opportunities for unaided idea generation should be built into any activity. Implementation should pause where child-safety protections, informed governance, trained facilitation, or reliable infrastructure are absent.

For policymakers, priority should be given to independent evaluation and equity-sensitive infrastructure rather than universal rollout. Funding should explicitly include rural, underserved, low-connectivity, disability, and linguistically diverse communities in co-design and comparative trials. Policies should address child data privacy, acceptable use, academic integrity, procurement transparency, and the preservation of non-AI alternatives. Until direct evidence demonstrates benefits in underserved settings, GenAI should not be presented as a proven democratizing intervention.

For technology developers, the core recommendation is designing purpose-built, child-centered GenAI tools for K-12 creative education incorporating algorithmic bias mitigation, robust privacy protection, age-appropriate content filtering, and simplified, intuitive prompt interfaces adapted to children's language and metacognitive skills (Fan et al., 2024; Yang et al., 2025). Developers should collaborate closely with developmental psychologists, educators, and creative experts to ensure tools align with children's creative development principles supporting not just idea generation but elaboration, refinement, and collaboration (Silvennoinen et al., 2024; Wang et al., 2025). They should also provide free, accessible educator resources to support effective integration and design educator dashboards that enable monitoring of children's GenAI use without compromising privacy.

For parents and communities, co-use and discussion can help children question AI outputs, distinguish AI-generated from personally developed ideas, and retain ownership of the creative process. However, these recommendations assume access, time, digital literacy, and safe tools that are not evenly distributed. Cross-sector collaboration should therefore include community organizations and underserved families as decision-makers, with adoption contingent on locally demonstrated benefit and safeguards rather than on generalized equity claims.

### 4.6 Limitations

This scoping review has several limitations. First, it included English-language sources and therefore may underrepresent non-English evidence. Second, the 24-source set is heterogeneous and contains 22 primary empirical studies plus two secondary syntheses; the latter were used only for context and cannot be treated as independent empirical confirmation. Third, peer review was not required. The corpus includes the Horowitz-Kraus et al. (2025) bioRxiv preprint, an arXiv preprint (Hu X. et al., 2025), an SSRN working paper (Silvennoinen et al., 2024), and an institutional report (Picton and Clark, 2024). Their findings require cautious interpretation and updating after formal publication. Fourth, no formal critical appraisal or risk-of-bias assessment was conducted, and the heterogeneity of designs and measures precluded meta-analysis; accordingly, this review cannot establish efficacy or comparative effectiveness. Fifth, positive-result and publication bias may be substantial in a rapidly developing field, particularly when null findings and unsuccessful implementations remain unpublished. Sixth, Google Scholar supplied 3,859 of 6,401 initial records. Its broad coverage improved sensitivity but introduced noise, unstable ranking, gray literature, and limited search reproducibility despite reporting the parenthesized query and search dates. Finally, the evidence is geographically concentrated and contains almost no direct research in rural or underserved communities. Equity and democratization claims are therefore hypotheses for future testing, not conclusions supported by the present corpus.

Future research should prioritize longitudinal and preregistered comparative studies; report effect sizes and null findings; replicate preliminary preprint results after peer review; and use validated creativity measures that distinguish AI-assisted output quality from independent creative capacity. Equity-focused studies should recruit rural, underserved, low-connectivity, disability, and linguistically diverse populations from the design stage. Only after benefits, harms, feasibility, and resource demands are directly evaluated in these settings should claims about democratization or scalable equitable adoption be advanced.

## 5 Conclusion

This systematic scoping review mapped 24 evidence sources, 22 primary empirical studies and two secondary syntheses, on GenAI and children's creative thinking. The literature suggests that supervised GenAI may help some children generate, express, or refine ideas, but it does not confirm efficacy, durable cognitive development, scalability, or equity. The evidence is heterogeneous, geographically concentrated, partly non-peer-reviewed, and unaccompanied by formal risk-of-bias appraisal or meta-analysis. GenAI should therefore be considered a conditional supplement to human-led creative learning, tested through cautious, age-appropriate, and locally evaluated use. Stronger longitudinal, comparative, and equity-focused research is required before broad adoption or democratization claims are warranted.

## Data Availability

The original contributions presented in the study are included in the article/supplementary material, further inquiries can be directed to the corresponding authors.
